# Generation of Q-switched Pulses in Thulium-doped and Thulium/Holmium-co-doped Fiber Lasers using MAX phase (Ti_3_AlC_2_)

**DOI:** 10.1038/s41598-020-66141-3

**Published:** 2020-06-08

**Authors:** H. Ahmad, A. A. Kamely, N. Yusoff, L. Bayang, M. Z. Samion

**Affiliations:** 1 Photonics Research Centre, University of Malaya, 50603 Kuala Lumpur, Malaysia; 2 Physics Dept., Faculty of Science, University of Malaya, 50603 Kuala Lumpur, Malaysia

**Keywords:** Optics and photonics, Lasers, LEDs and light sources, Optical materials and structures, Optical physics

## Abstract

A MAX phase Ti_3_AlC_2_ thin film is demonstrated as a saturable absorber (SA) to induce Q-switching in the 2.0 μm region. The Ti_3_AlC_2_ thin film is sandwiched between two fiber ferrules and integrated into thulium doped fiber laser (TDFL) and thulium-holmium doped fiber laser (THDFL) cavities. Stable Q-switched pulses are observed at 1980.79 nm and 1959.3 nm in the TDFL and THDFL cavities respectively, with repetition rates of 32.57 kHz and 21.94 kHz and corresponding pulse widths of 2.72 μs and 3.9 μs for both cavities. The performance of the Ti_3_AlC_2_ based SA for Q-switching operation indicates the high potential of other MAX phase materials to serve as SAs in future photonics systems.

## Introduction

Q-switched fiber lasers are highly desired laser sources for a wide number of applications due to their potential to produce high energy pulses for use in applications such as sensors^1^, micromachining^2,3^ and medical systems^4–6^. Recently, Q-switching in the 2 micron region has received increasing interest due to its various applications such as optical communications^7^, remote sensing^8^ and in particular for biological and medical applications^9^. Q-switched lasers operating at 2.0 µm have been demonstrated for various applications such as the treatment of skin discoloration due to melasma pigmentation^10–12^ by multiple passes with a large spot size laser^13^, as well as for the removal of tattoos^14–19^. Additionally, Q-switched fiber lasers operating at this region are also highly suitable for micromachining applications such as engraving^20–24^, cutting^25,26^, micro-welding^27,28^ and drilling^29,30^.

While erbium doped fibers (EDFs) are the most commonly used gain medium in fiber lasers^31^, efficient operation in the 2.0 µm region is instead achieved by the use of  thulium (TDF)^32^, holmium (HDF)^33^ and thulium-holmium^34^ doped fibers (THDF). Of the three, the TDF is the most popular choice due to its broad emission from 1.6 to 2.2 micron^35^. As such, thulium doped fiber lasers (TDFLs) and thulium-holmium doped fiber lasers (THDFLs) are typically used to develop high power and stable ultra-short pulses in the 2.0 µm region with wideband wavelength tuning^36–39^. Originally, these pulses are generated by active methods, such as acoustic-optic modulators (AOMs)^40^ or electric-optic modulators (EOMs)^41^, though these cavities suffer from limitations due to their bulk, complex operation and expensive fabrication^42,43^. As such, passive method have now become the focus of research efforts, due to their significantly simpler design, lower cost and easier fabrication^44,45^.

Of the may techniques to generate passive Q-switching in a fiber laser cavity, saturable absorbers (SAs) are the most common^46^. SAs are typically classified as either artificial and real, with artificial SA typically formed by exploiting various nonlinear optical phenomenon such as nonlinear polarization rotation (NPR)^47^ and nonlinear optical loop mirrors (NOLMs)^48,49^. Real SAs on the other hand are materials based, such as semiconductor saturable absorber mirrors (SESAMs)^50^, carbon nanotubes (CNTs)^51,52^, graphene^53,54^, topological insulators (TIs)^55,56^, black phosphorus (BP)^57,58^, transition metal dichalcogenides (TMDs)^59^, transition metal oxides (TMOs)^60,61^ and alcohol^62^. Recently as well, the discovery of the optical properties of MXene has made it a potential SA material^63,64^. The synthesis of MXene requires an etching of a MAX phase, commonly by using a combination of strong fluoride ion (F^−^) etching solutions, hydrochloric acid (HCl) and lithium fluoride (LiF)^65^. The fabrication process of the MAX phase material would therefore minimize time and cost as it does not require the etching process and thus the need to use strong etching solutions. Furthermore, MAX phase composes of layered ternary transition-metal carbides that have both metal and ceramic properties, which could be useful for high-temperature applications^66^. The MAX phase is also stiff, oxidation resistant and lightweight in terms of its ceramic properties, while the metallic properties causes it to have good thermal and electrical conductivity, machinability, damage tolerance and thermal shock resistance^67,68^.

In this work, Q-switched pulse generation in  TDFL and THDFL cavities using MAX phase thin film Ti_3_AlC_2_ based SAs are proposed and demonstrated. The fabrication of MAX phase Ti_3_AlC_2_ as an SA saves one step in comparison to the fabrication of MXene while still having the potential to produce Q-switched pulses. The proposed SA has not been widely used and never been demonstrated in 2 μm region to the best of author’s knowledge. Hence, the potential of Ti_3_AlC_2_ to realize Q-switched pulse in 2 μm region is investigated in two different gain mediums where the proposed laser would complement the current fiber laser technology.

## Characterizations of Ti_3_AlC_2_-PVA Film

**Figure 1:**
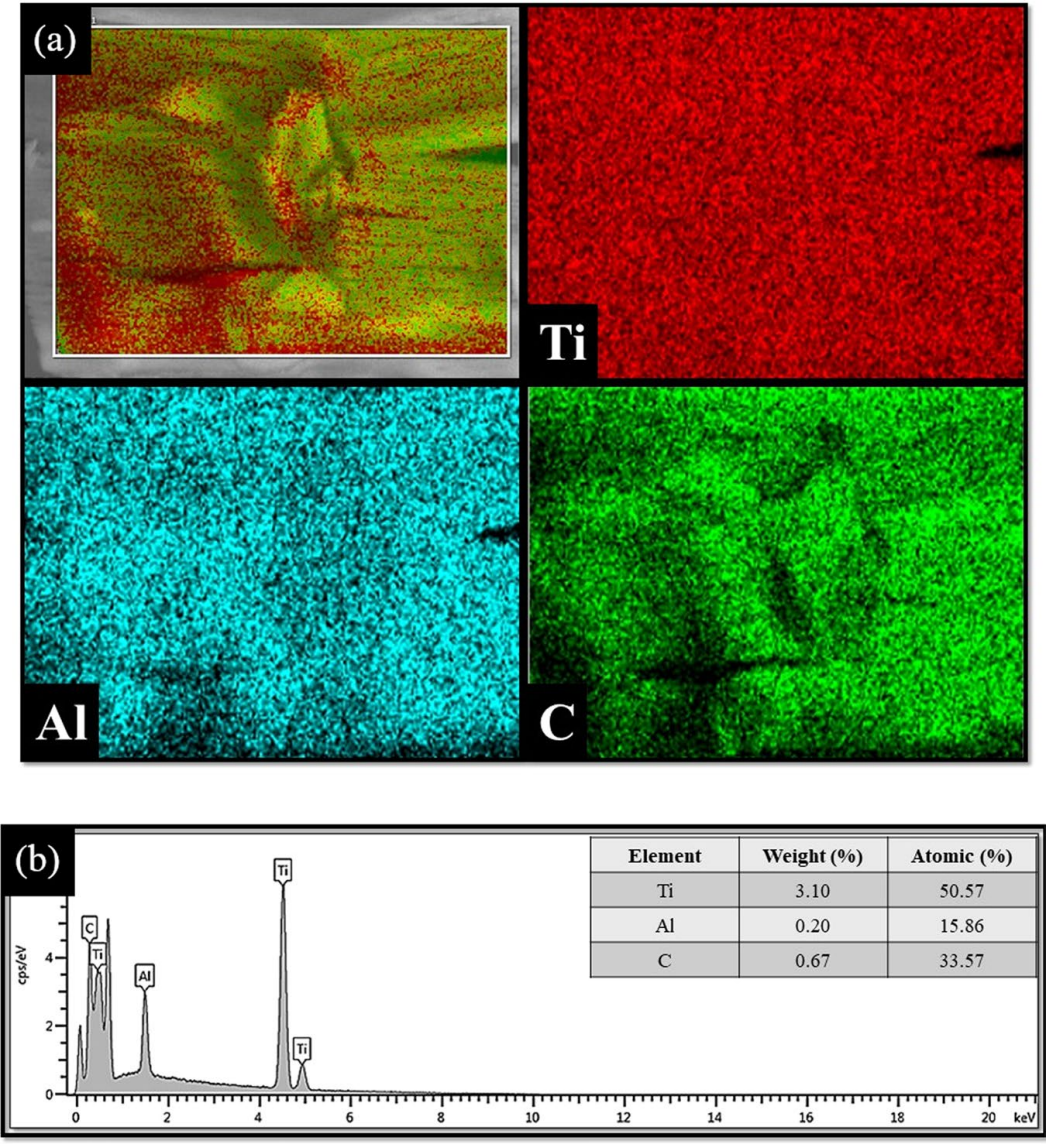
(**a**) EDX elemental mapping of Ti_3_AlC_2_ with its corresponding **(b)** EDX spectrum.

The elemental composition of the Ti_3_AlC_2_ MAX phase is determined by energy dispersive X-ray (EDX) analysis and the results are presented in Fig. 1. As shown in Fig. 1(a), the EDX elemental mapping clearly shows titanium (Ti), aluminum (Al), and carbon (C) elements and therefore indicates the successful preparation of the Ti_3_AlC_2_ MAX phase. The three elements are distributed homogeneously on the surface of Ti_3_AlC_2_. To further confirm the chemical composition of the Ti_3_AlC_2_ MAX phase, EDX spectroscopy is undertaken and the results shown in Fig. 1(b). The EDX spectrum of Ti_3_AlC_2_ shows strong signals corresponding to its main elements including Ti, Al, and C. The presence of these elements with the absence of other impurities elements verify the formation of the Ti_3_AlC_2_ MAX phase.

**Figure 2:**
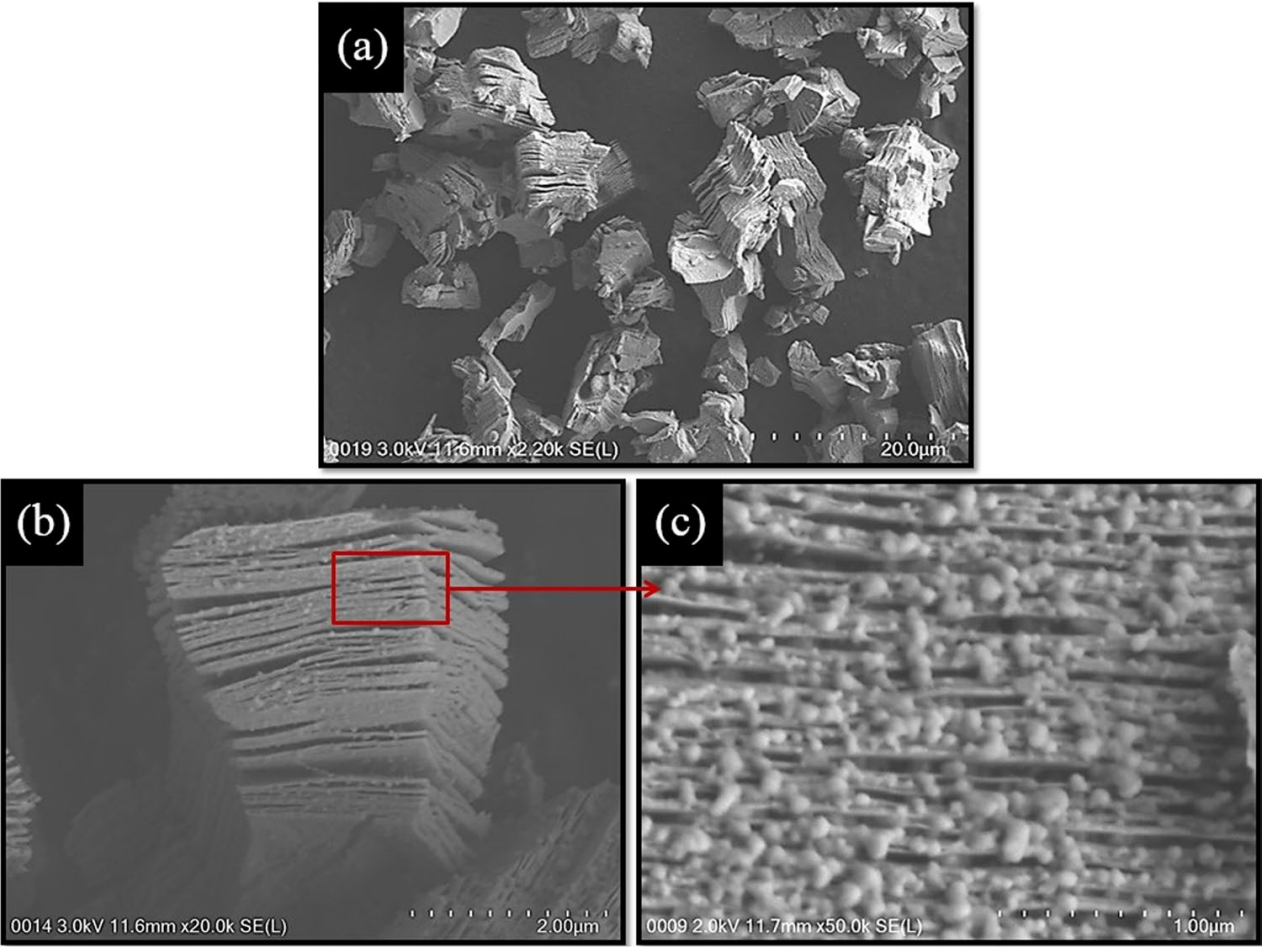
FESEM images of Ti_3_AlC_2_ MAX phase obtained under different magnification: (**a**) 2.2 k, **(b)** 20.0 k, and **(c)** 50.0 k x magnification.

The morphological properties of Ti_3_AlC_2_ are observed by field emission scanning electron microscope (FESEM) analysis using a Hitachi SU8220 FESEM. Figure 2 shows the FESEM images of the Ti_3_AlC_2_ MAX phase acquired at different magnifications. It can be seen that the Ti_3_AlC_2_ MAX phase is built up from alternating Ti-C and Al layers, forming a lamellar structure as can be seen in Fig. 2(a,b). From the high magnification image given in Fig. 2(c), it can be clearly observed that all the layers are tightly packed together as a result of the metallic bond between each layers that connects them.

**Figure 3:**
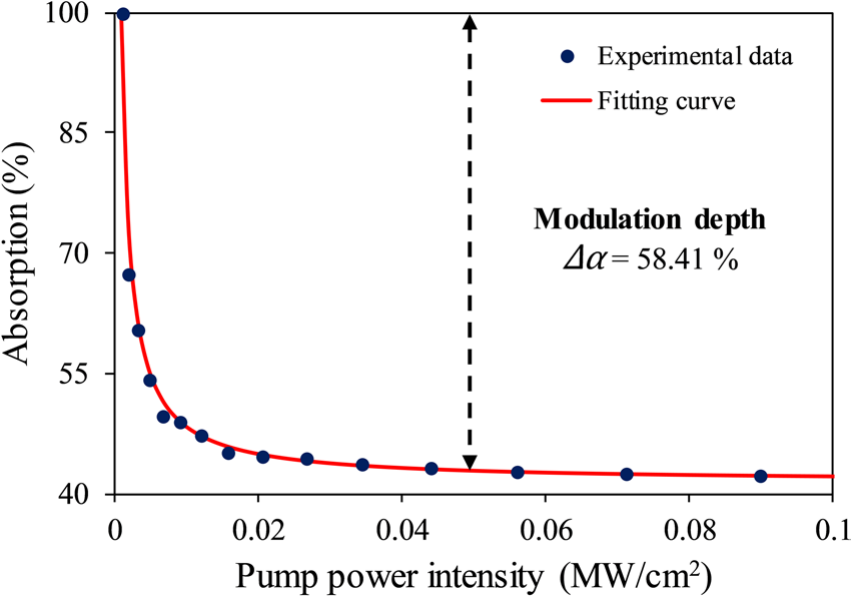
Nonlinear saturable absorption curve of Ti_3_AlC_2_.

A mode-locked laser operating at 1566 nm with a repetition rate of 27.7 MHz and a pulse width of 0.70 ps is used to measure the nonlinear response of the Ti_3_AlC_2_ by the balanced twin-detector method. A variable attenuator is introduced in the measurement system to control the input power into Ti_3_AlC_2_. It must be noted that in this work a mode-locked laser output at 2.0 μm could not be used due to the lack of necessary equipment. The nonlinear saturable absorption curve of the Ti_3_AlC_2_ MAX phase is given in Fig. 3.

The nonlinear saturable absorption curve of the Ti_3_AlC_2_ is taken by fitting the intensity-dependent absorption equation into the measured data:

$$\alpha (I)=\frac{\Delta \alpha }{\left(1,+,\frac{I}{{I}_{sat}}\right)}+{\alpha }_{ns}$$

The parameters Δ*α*, *I*_*Sat*_ and *α*_*ns*_ are modulation depth, saturation intensity and non-saturable loss, respectively. The modulation depth and non-saturable loss of Ti_3_AlC_2_ are computed to be 58.41% and 41.59%, respectively.

## Experimental setup

**Figure 4:**
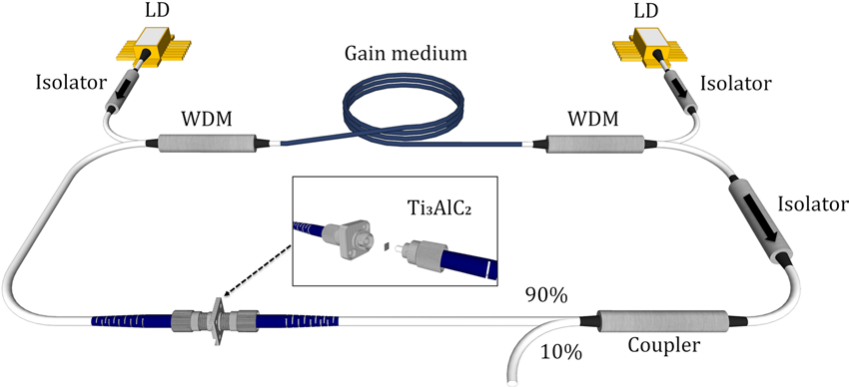
Schematic of laser cavity for Q-switched pulse generation in TDFL and TDHFL. This figure was drawn using SketchUp Make 2017 (Basic), Software Version: Windows 64-bit 17.2.2555, available at https://www.sketchup.com/download/all.

The cavity setup for Q-switched TDFL and THDFL are given in Fig. 4. It is imperative to note that for both the TDFL and THDFL, the overall cavity is the same, with only the gain medium changed. For the cavity, two Princeton Lightwave Inc PSL 450 1550 nm laser diodes (LDs) with maximum powers of 240 mW each are used to optically pump the 4 m long TmDF200 TDF that is commercially available from OFS Inc. The TDF has a cutoff wavelength of 1350 nm and an absorption coefficient of about 200 dBm^−1^ at 790 nm and 22 dBm^−1^ at 1550 nm. The TDF is optically pumped through the 1550 nm port of 1550/2000 nm wavelength division multiplexers (WDMs), where 1550 nm isolators (ISOs) are used between LDs and ports of the WDMs to prevent any back-reflections from damaging the LDs.

A 2000 nm ISO is placed after 2000 nm port of the WDM to ensure the unidirectional propagation of the signal in the cavity. A 90:10 optical coupler (OC) is connected to the ISO output to extract 10% from the intra-cavity light for analysis. The 90% port of the OC continues to travel in the cavity, where it encounters the Ti_3_AlC_2_ based SA which induces pulsing in the propagating signal. The SA is sandwiched between two fiber connectors and held by an FC/PC connector to form the SA assembly. The output from the SA is then connected to the 2000 nm port of the WDM to complete the optical cavity. The incorporation of a polarization controller (PC) is not needed as Q-switched pulses can be observed once the MAX phase Ti_3_AlC_2_ film is incorporated into the laser cavity, without the need of a further optimization of the polarization states in the cavity.

For the configuration of the THDFL, the same cavity setup is used, with the only difference being the replacement of the TDF with a 1.5 m long CorActive TH512 thulium-holmium doped fiber as the gain medium. The THDFL has a cut-off wavelength of between 1650 nm to 1750 nm and an absorption coefficient of 120 dBm^−1^ at around 790 nm as well as 15 dBm^−1^ at 1550 nm.

## Results and Discussion

### Thulium-doped fiber laser (TDFL) with Ti_3_AlC_2_

**Figure 5:**
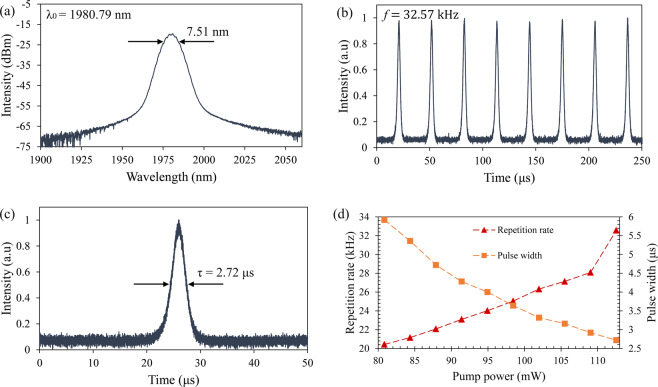
Pulse characteristic of Q-switched TDFL. (**a**) Optical spectrum, (**b**) pulse train and (**c**) single pulse profile at pump power of 112.55 mW. (**d**) Pulse repetition rate and pulse width against pump power.

A pure PVA film is first tested in both the TDFL and THDFL cavities to observe any pulse lasing existence, but only continuous wave (CW) lasing is observed even when the pump power is increased to its maximum. This confirms that the Q-switching effect is induced by the Ti_3_AlC_2_. Furthermore, it is also proven that there is no absorption by the pure PVA film in the UV spectrum^69^, thus confirming that the PVA film has no role in inducing the Q-switched output other than being a host material. When configured as a TDFL, CW lasing is obtained at a pump power of 75.56 mW and a stable Q-switched pulse train is achieved when the pump power reaches 80.84 mW. A maximum pump power of 112.55 mW is observed for the Q-switched operation of the TDFL, as any higher pump powers will result in significant fluctuations and instabilities of the pulses. This is attributed to the SA beginning to suffer the effects of optical damage but has not yet experienced any significant damage as lowering the pump power results in a stable pulse train being obtained once again. This implies that the SA could only withstand a corresponding power intensity of about 0.44 MW/cm^2^ before the Q-switching performance started to degrade. The Q-switched output has an average output power of 0.67 mW to 1.43 mW for a pump power of 80.84 mW to 112.55 mW. Figure 5(a) shows the optical spectrum of the Q-switched pulses at a pump power of 112.55 mW with a center wavelength of 1980.79 nm, where the full width at half maximum (FWHM) of the spectrum is 7.51 nm. The laser appears broader than usual, and this is attributed to the self-phase modulation (SPM) effect in the cavity that takes place when a high intensity laser propagates through the fiber.

In Fig. 5(b,c), the pulse train and single pulse profile are taken in the time domain using an OSC at a maximum pump power of 112.55 mW. The pulse train shows a uniform pulse intensity with a repetition rate of 32.57 kHz and the single pulse shows a pulse width of 2.72 μs. The pulses do not fluctuate significantly throughout the measurement, with uniform shape and pulse intensity. The behaviors of pulse repetition rate and pulse width are observed by varying the pump power from 80.84 mW to 112.55 mW. The repetition rate and the pulse width are 20.44 kHz to 32.57 kHz and 5.92 μs to 2.72 μs, respectively as shown in Fig. 5(d), where the repetition rate and the pulse width are inversely proportional to each other as the pump power increases. The pulse width decreases at a constant rate until the maximum pump power value shows that the SA has not reached its saturation limit yet. Increasing the pump power above the maximum value will cause the degradation of the SA and result in a higher Q-switching threshold of the SA initially, and if continued to operate above the maximum pump power the possible failure of the SA.

**Figure 6:**
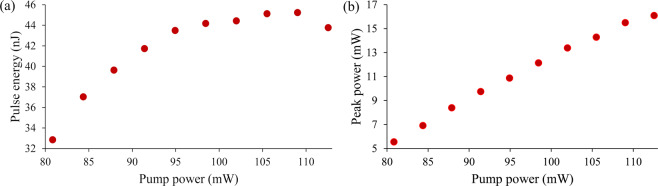
Output trend of TDFL (**a**) pulse energy and (**b**) peak power against pump power.

**Figure 7:**
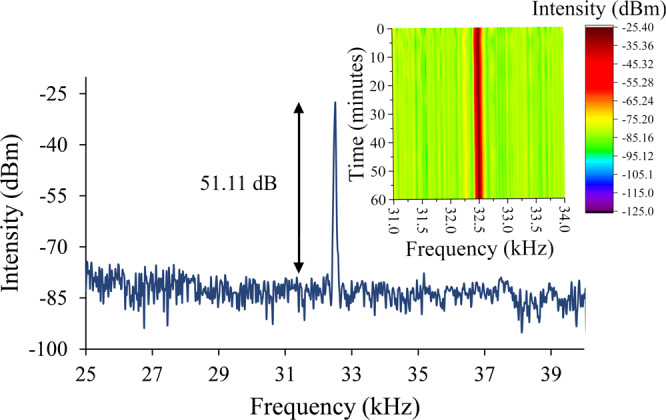
RF spectrum stability of TDFL at 112.55 mW over 60 minutes.

In Fig. 6, the pulse energy as well as peak power are calculated over the Q-switching pump power range. The pulse energy and the peak power in Fig. 6(a,b) show the highest pulse energy generated at the pump power of 109.03 mW and the highest peak power at the pump power of 112.55 mW, which are 45.23 nJ and 15.49 mW respectively. In addition, a signal-to-noise ratio (SNR) at 32.57 kHz in the frequency domain is  measured by using RFSA at a pump power of 112.55 mW as shown in Fig. 7. The output pulse has an average SNR of 51.11 dB, which is comparable to other Q-switched lasers that have SNRs of more than 40 dB^70^. The stability of the peak is measured for 60 minutes at 5 minute intervals. The peak shows no significant fluctuations throughout the observation period, thus showing a stable Q-switched system. The same can be said about the laser’s output power, as any fluctuation in the laser’s output power would affect the intensity of the Q-switched pulses and hence will also be reflected in the intensity of the RF spectrum.

### Thulium-holmium doped fiber laser (THDFL)

**Figure 8:**
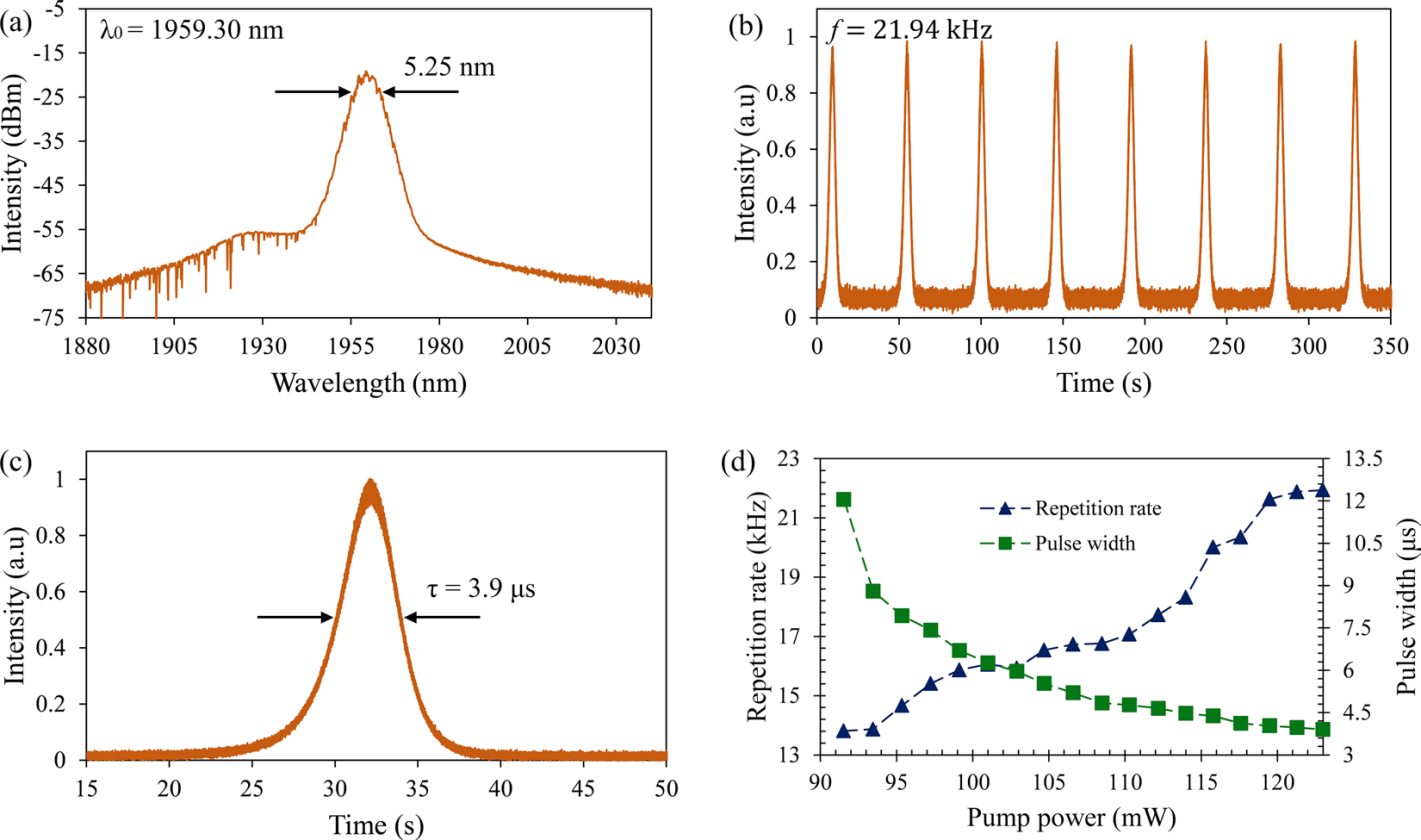
Pulse characteristic of Q-switched THDFL. (**a**) Optical spectrum, (**b**) pulse train and (**c**) single pulse profile at pump power of 123 mW. (**d**) Pulse repetition rate and pulse width against pump power.

In the THDFL configuration, CW lasing is obtained at a pump power of 85.8 mW before a stable Q-switched pulse train is obtained when the pump power reaches 91.54 mW. It is worth to note that the Q-switching threshold is lower in the TDFL as compared to that of THDFL. This is due to the TDF having a higher absorption at 1550 nm as compared to the THDF, which allows the TDF to absorb the pump signal more efficiently. The pump power is then gradually increased until a maximum pump power of 123 mW, above which major fluctuations and instabilities are observed in the pulses due to it nearing the SA’s saturation limit. The average output power was measured to be 0.48 mW to 1.29 mW at a pump power range of 91.54 mW to 123 mW. The optical spectrum of the Q-switched at a pump power of 123 mW has a center wavelength of 1959.3 nm and FWHM of 5.25 nm as shown in Fig. 8(a). As with the case of the TDFL, the high intensity lasing in the cavity induces the SPM effect and results in the broadening of the laser spectrum.

The pulse train and single pulse profile at maximum pump power of 123 mW are taken in the time domain using an OSC as shown in Fig. 8(b,c). A uniform pulse intensity with a repetition rate of 21.94 kHz and a pulse width of 3.9 μs are shown in the pulse train and single pulse profile, respectively. Throughout the measurement, the pulses maintain uniform shape and pulse intensity, indicating a stable Q-switched laser output. In Fig. 8(d), the pump power is varied from 91.54 mW to 123 mW to study the behavior of pulse repetition rate and pulse width. The repetition rate and the pulse width are 13.81 kHz to 21.94 kHz and 12.05 μs to 3.9 μs, respectively, and are proportionally inversed to each other as the pump power increases. The pulse width has a sudden drop from 12.05 μs to 8.8 μs when the pump power increases from 91.54 mW to 93.46 mW, but gradually decreases above a pump power of 93.46 mW until the maximum value. The exponential decline of the pulse width shows that the SA is nearing its saturation limit, especially for a pulse width from 4.12 μs to 3.9 μs which appears to reduce only minimally.

**Figure 9:**
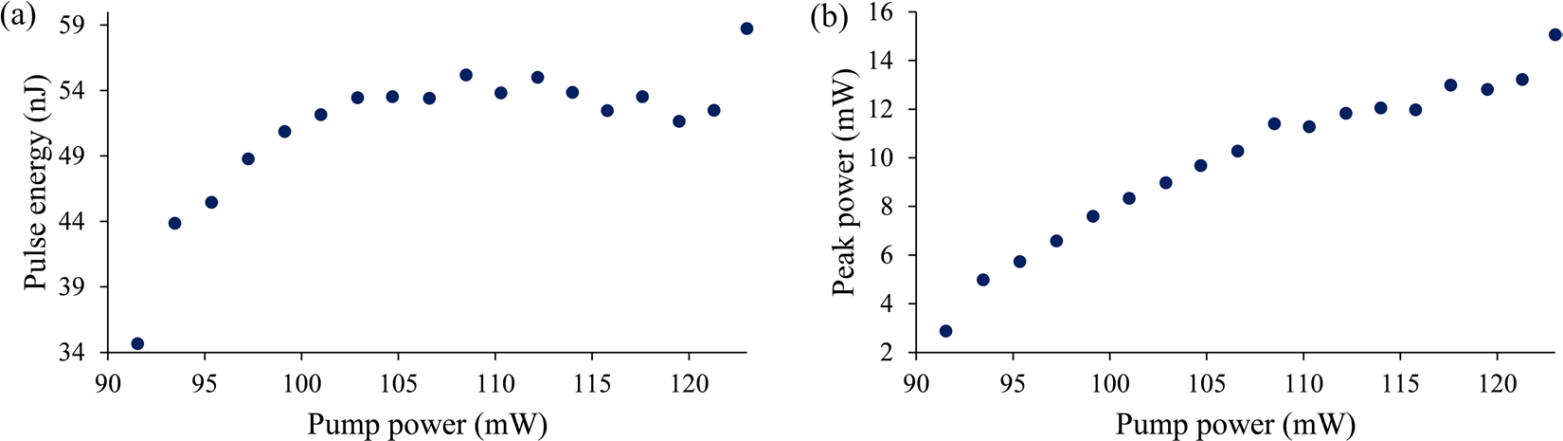
Output trend of THDFL (**a**) pulse energy and (**b**) peak power against pump power.

**Figure 10:**
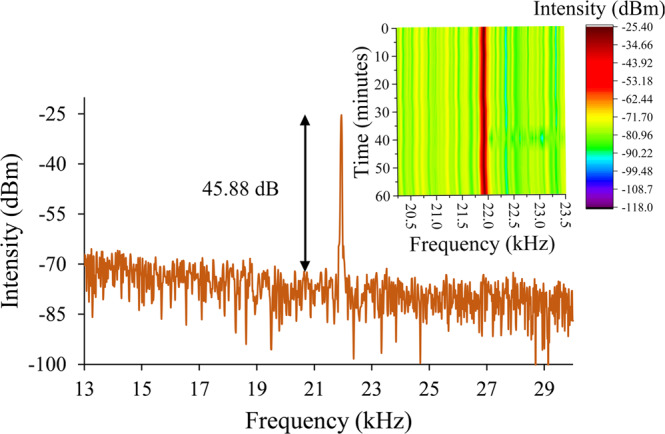
RF spectrum stability of THDFL at 123 mW over 60 minutes.

Pulse energy and peak power are calculated with respect to the Q-switched pump power range. The pulse energy and the peak power in Fig. 9(a,b) show the highest pulse energy generated at the pump power of 123 mW and the highest peak power at the pump power of 123 mW, which are 58.72 nJ and 15.06 mW, respectively. Furthermore, the pulse peak in frequency domain is also measured using a RFSA to verify the phase noise and to evaluate the stability of the system as shown in Fig. 10. The pulse peak at 21.94 kHz corresponds well with the repetition rate of the pulses at maximum pump power and the average SNR of the peak is measured to be 45.88 dB, which is notably high. The test for peak stability is taken for 60 minutes, where every measurement is taken at 5 minutes interval. A stable Q-switched system is displayed as no major fluctuation occurs at the peak throughout the period.

The cavity is also tested without the incorporation of the Ti_3_AlC_2_ for both configurations. In this case, both configurations show no sign of Q-switching at any pump power level, hence confirming the thin film Ti_3_AlC_2_ is responsible solely for inducing the Q-switching effect. In addition to that, no mode-locking phenomenon is observed in both of the laser cavities using the Ti_3_AlC_2_ SA. We believe this is due to the high non-saturable loss of the SA as well as the high intracavity loss in the laser cavity. Mode-locking operation could be obtained by using a high-quality SA with low non-saturable loss^71^, by controlling the loss of the cavity as well as by adjusting the polarization states of the intracavity signal using a PC. The performance of the Q-switched lasers in 2.0 μm region with different thin films are compared in Table 1.

**Table 1:** Comparison of all-fiber Q-switched lasers operating in 2.0 μm region.

Saturable absorber	Operation wavelength (nm)	Pulse width (μs)	Repetition rate (kHz)	Maximum pulse energy (μJ)	Reference
TiO_2_	1935	3.91–1.91	30.12–36.96	0.3	^73^
MoS_2_	2032	2.50–1.76	33.60–48.10	1	^74^
MoSe_2_	1924	16.00–5.50	14–21.80	0.042	^75^
Ti_3_AlC_2_	1980 (TDFL)	5.92–2.72	20.44–32.57	0.045	[This work]
	1959 (THDFL)	12.05–3.90	13.81–21.94	0.058	

From the table, it can be seen that the minimum pulse width and maximum repetition rate of TiO_2_ and MoS_2_ surpass both MoSe_2_ and Ti_3_AlC_2_, which are 1.91 μs and 1.76 μs as well as 36.96 kHz and 48.10 kHz, respectively. Furthermore, the maximum pulse energy generated from TiO_2_ and MoS_2_ also exceed that of MoSe_2_ and Ti_3_AlC_2_, which are 0.3 μJ and 1 μJ, respectively. Nevertheless, the performance of Ti_3_AlC_2_ is comparable to MoSe_2_, where the minimum pulse width and the maximum repetition rate of MoSe_2_ are 5.50 μs and 21.80 kHz, which are both below Ti_3_AlC_2_ of 2.72 μs and 32.57 kHz for TDFL and 3.90 μs and 21.94 kHz for THDFL. The maximum pulse energy of MoSe_2_ is 0.042 μJ, slightly lower than Ti_3_AlC_2_ in TDFL and THDFL, which are 0.045 μJ and 0.058 μJ, respectively. Although current works on the MAX phase Ti_3_AlC_2_ thin film are limited, it can still produce Q-switched pulses with considerably good performance. Furthermore, the optical damage threshold limitation can also be overcome by having the SA materials interact with the TDFL or THDFL through the evanescent wave of the propagating beam, as opposed to being directly exposed to the signal. This can be achieved by using a tapered fiber, with the material being sprayed at the waist of the tapered fiber^72^, and can realize higher output powers from both cavities.

## Methods

### Preparation of Ti_3_AlC_2_-PVA film

The Ti_3_AlC_2_ powder is purchased from Laizhou Kai Kai Ceramic Material Co. Ltd. and polyvinyl alcohol (PVA) with M_W_ ~ 31,000 is obtained from Sigma Aldrich. In order to form the Ti_3_AlC_2_-PVA film, solution casting is employed with the PVA as the host polymer. Approximately 100 mg of Ti_3_AlC_2_ powder is ultrasonicated for 16 hours in 20 mL of deionized water (DIW) to form a homogeneous solution with an initial concentration of 5 mg/mL. Any undissolved powder is separated from the homogeneous dispersion by centrifugation at 3000 rpm for 15 minutes, and the supernatant is collected for further use. At the same time, the PVA solution with a concentration of 5 mg/mL is prepared by dissolving 100 mg of the PVA powder in 20 mL of DIW while being stirred at 60 °C until the powder is totally dissolved. 10 mL of each solution is then mixed together in a beaker. The mixture is stirred for 2 hours while on a hot plate at 60 °C before being poured into a petri dish and put it in an over at 60 °C for process.

### Laser characterization

The optical spectrum of the Q-switched laser is analyzed using a Yokogawa AQ6370B optical spectrum analyzer (OSA) with a wavelength range from 1200 nm to 2400 nm, while a 10 GHz Newport 818-BB-51F photodetector (PD) with a responsivity of 0.95 A/W at 2000 nm is used for time and frequency domain analysis. A Keysight DSOX3102T oscilloscope (OSC) with a bandwidth of 1 GHz and an Anritsu MS2683A radio frequency spectrum analyzer (RFSA) are used to monitor the output in time and frequency domain, respectively.

## Conclusion

This work successfully demonstrates Q-switched operation at the 2.0 μm region in TDFL and THDFL incorporating thin film Ti_3_AlC_2_ as a new SA, capable of producing stable pulse with kilohertz repetition rates and microsecond pulse widths. The Q-switched pulses operating wavelength for TDFL and THDFL are observed at 1980.79 nm and 1959.3 nm, respectively. Furthermore, maximum repetition rates of 32.57 kHz and 21.94 kHz as well as minimum pulse widths of 2.72 μs and 3.9 μs are observed at maximum pump power of 112.55 mW and 123 mW for the TDFL and THDFL, respectively. Besides that, uniform pulse intensity and stable pulses are shown in each laser output, with the average SNRs of 51.11 dB and 45.88 dB for TDFL and THDFL, respectively. The MAX phase Ti_3_AlC_2_ based SA can complement the current application of fiber laser technology.
